# Brahma Related Gene 1 (Brg1) Regulates Cellular Cholesterol Synthesis by Acting as a Co-factor for SREBP2

**DOI:** 10.3389/fcell.2020.00259

**Published:** 2020-05-15

**Authors:** Zhiwen Fan, Ming Kong, Min Li, Wenxuan Hong, Xiangshan Fan, Yong Xu

**Affiliations:** ^1^Department of Pathology, Nanjing Drum Tower Hospital, Nanjing University Medical School, Nanjing, China; ^2^Key Laboratory of Targeted Intervention of Cardiovascular Disease and Collaborative Innovation Center for Cardiovascular Translational Medicine, Department of Pathophysiology, Nanjing Medical University, Nanjing, China; ^3^Department of Clinical Medicine and Laboratory Center for Experimental Medicine, Jiangsu Health Vocational College, Nanjing, China; ^4^Institute of Biomedical Research, Liaocheng University, Liaocheng, China

**Keywords:** transcriptional regulation, epigenetics, hepatocyte, cholesterol synthesis, SREBP2, BRG1, histone demethylase

## Abstract

Hepatocyte is a hub for cholesterol metabolism. Augmented synthesis of cholesterol in the liver is associated with hypercholesterolemia and contributes to the pathogenesis of a host of cardiovascular and metabolic diseases. Sterol response element binding protein 2 (SREBP2) regulates hepatic cholesterol metabolism by activating the transcription of rate-limiting enzymes in the cholesterol biosynthesis pathway. The underlying epigenetic mechanism is not well understood. We report here that mice with hepatocyte-specific knockout (CKO) of Brg1, a chromatin remodeling protein, exhibit reduced levels of hepatic cholesterol compared to the wild type (WT) littermates when placed on a high-fact diet (HFD) or a methionine-and-choline-deficient diet (MCD). Down-regulation of cholesterol levels as a result of BRG1 deficiency was accompanied by attenuation of cholesterogenic gene transcription. Likewise, BRG1 knockdown in hepatocytes markedly suppressed the induction of cholesterogenic genes by lipid depletion formulas. Brg1 interacted with SREBP2 and was recruited by SREBP2 to the cholesterogenic gene promoters. Reciprocally, Brg1 deficiency dampened the occupancies of SREBP2 on target promoters likely through modulating H3K9 methylation on the cholesterogenic gene promoters. Mechanistically, Brg1 recruited the H3K9 methyltransferase KDM3A to co-regulate pro-cholesterogenic transcription. KDM3A silencing dampened the cholesterogenic response in hepatocytes equivalent to Brg1 deficiency. In conclusion, our data demonstrate a novel epigenetic pathway that contributes to SREBP2-dependent cholesterol synthesis in hepatocytes.

## Introduction

Cholesterol is an important bioactive lipid that plays a wide range of physiological and pathophysiological roles. As a major component of biomembranes, cholesterol regulates trans-membrane signaling events by modulating membrane fluidity (Sengupta and Chattopadhyay, 2015; Legler et al., 2017; Murrell-Lagnado, 2017). Cholesterol homeostasis is essential for development (Hussain et al., 2019), reproduction (Sedes et al., 2018), and cognitive function (Chang et al., 2017). Recent studies have suggested that cholesterol may also contribute to innate immunity and host defense (Reboldi and Dang, 2018). On the other hand, excessive cholesterol synthesis is associated with hypercholesterolemia and a host of cardiovascular and metabolic diseases. Hepatocytes are the primary source of cholesterol production. Over-production of cholesterol can often be attributed to up-regulation of the enzymes involved in its biosynthetic pathway in the liver. As a matter of fact, statins, widely used to treat dyslipidemia and coronary heart disease (CHD), target 3-hydroxy-3-methylglutaryl-CoA reductase (HMGCR), a rate-limiting enzyme for cholesterol biosynthesis (Levy et al., 1993).

Lipogenesis, including cholesterogenesis, in hepatocytes is programmed, at the transcription level, by a network of transcription factors (Lee et al., 2019). Sterol response element binding protein (SREBP) family of transcription factors are master regulators of lipid synthesis. First identified by the Brown and Goldstein laboratory, the SREBP family includes three members: SREBP1a, SREBP1c, and SREBP2 (Horton et al., 2002b). Of the three SREBP isoforms, SREBP1c and SREBP2 are predominantly expressed by most animal tissues (Shimomura et al., 1997). By virtue of programming cellular metabolism, SREBP proteins play key roles in the pathogenesis of human diseases (Wei et al., 2018; Wen et al., 2018; Gobel et al., 2019; Yin et al., 2019). All (mature) SREBPs can be categorized as basic helix-loop-helix (bHLH) transcriptional factors. Structural and functional studies have found that SREBP2 and SREBP1a both possess longer trans-activation domains than SREBP1c and therefore more potent transcriptional activators (Shimano et al., 1997a). Investigations with transgenic animal models have further revealed that SREBP1c is primarily responsible for fatty acid synthesis *in vivo* whereas SREBP2 mainly orchestrates cholesterogenesis (Horton et al., 2002a). SREBP2 promotes cholesterol synthesis by directly activating the transcription of genes encoding key enzymes in the cholesterogenic pathway including *HMGCR*, low-density lipoprotein receptor (*LDLR*), 3-hydroxy-3-methylglutaryl-CoA synthase 1 (*HMGCS1*), and squalene monooxygenase (*SQLE*) (Howe et al., 2017).

Transcriptional regulation in mammalian cells is dictated by the epigenetic machinery that includes various histone and nucleotide modifying enzymes, histone variants, non-coding regulatory RNAs, and ATPase-dependent chromatin remodeling complexes (Goldberg et al., 2007). Brahma (BRM) and brahma related gene 1 (BRG1) are core components of mammalian SWI/SNF complex providing the ATPase activity (Hargreaves and Crabtree, 2011). BRG1-containing and BRM-containing complexes are mutually exclusive although their functional redundancies are not entirely clear. Unlike BRM, which is dispensable for embryonic development, BRG1 deficiency causes developmental arrest in mice (Bultman et al., 2000). In adults, BRG1 alteration has been associated with carcinogenesis and cardiovascular diseases (Hang et al., 2010; Wu et al., 2017; Li et al., 2018e; Zhang et al., 2018). Previously, we have shown that BRG1 contributes to the pathogenesis of non-alcoholic steatohepatitis (NASH), an emerging cause for hepatocellular carcinoma and cirrhosis, by regulating hepatic inflammatory response (Tian et al., 2013). In addition, hepatocyte conditional BRG1 deletion in mice attenuates SREBP1c-dependent fatty acid synthesis in the liver (Li et al., 2018b). These observations prompted a question as to whether, and if so, how BRG1 might regulate SREBP2-mediated cholesterol biosynthesis. Our data as reported here confirm that BRG1 plays an essential for the trans-activation of cholesterogenic genes in hepatocytes by acting as an epigenetic co-factor for SREBP2.

## Materials and Methods

### Animals

All animal procedures were reviewed and approved by the intramural Committee on Ethical Conduct of Animal Studies of Nanjing Medical University and in accordance with the NIH Guidelines for the Care and Use of Laboratory Animals. *Smarca4*-Flox mice (Li et al., 2018a) were crossed to *Alb*-Cre mice (Model Animal Research Institute, Nanjing, China) to obtain hepatocyte conditional Brg1 knockout (HepcKO) mice. 6–8 week-old male HepcKO mice and their wild type (WT) littermates were fed on a high-fat high-carbohydrate (HFHC) diet (Purchased from Research Diets, cat# D12492. Protein: 20% Kcal, Fat: 60% Kcal, Carbohydrate: 20% Kcal, Energy density: 5.21 Kcal/g) for 16 weeks to induce steatosis as previously described (Fan et al., 2017). Alternatively, the mice were fed on a methionine-and-choline-deficient (MCD) diet (Purchased from Research Diets, cat# A06071302. Protein: 18% Kcal, Fat: 62% Kcal, Carbohydrate: 21% Kcal, Energy density: 5.21 Kcal/g) for 8 weeks to induce steatosis.

### Cell Culture, Treatment, and Transfection

HepG2 cells were maintained in DMEM (Thermo Fisher, cat# 11965118) supplemented with 10% FBS (Thermo Fisher, cat# 10100147). To induce SREBP2 activity, cells were cultured in the LDM1 media containing lipid-depleted fetal bovine serum (Biowest, cat# S181L). Alternatively, cells were cultured in LDM2 media containing 0.1% bovine serum albumin (Sigma, cat# A2153) and 1% insulin-transferin-selenium (Thermo Fisher, cat# 41400045). The sequences for siRNAs are: siBRG1#1, AACATGCACCAGATGCACAAG; siBRG1#2, GCCCATGGAGTCCATGCAT; siSREBP2, CCCAUAAUAUC AUUGAGAA; siKDM3A#1, GACATGTGGTAATTCTGCAAGA ATT; siKDM3A#2, TAAATGCTTCACAATCAAAGC. Transient transfection was performed with Lipofectamine 2000 (Thermo Fisher, cat# 11668030). Cells were harvested 48 h after transfection.

### RNA Isolation and Real-Time PCR

RNA was extracted with the RNeasy RNA isolation kit (Qiagen, cat#74106) as previously described (Li et al., 2018c, 2019b). Reverse transcriptase reactions were performed with 1 μg total RNA as previously described using a SuperScript First-strand Synthesis System (Thermo Fisher, cat# 18091050) (Zeng et al., 2018). Real-time qPCR reactions were performed in triplicate wells on an ABI STEPONEPlus (Life Tech). The sequences for the primers are: human *BRG1*, 5′-GAGGAGGTCCGGCAGAAGAAATC-3′ and 5′-TTCTTCTGCTTCTTGCTCTC-3′; human *KDM3A*, 5′-GAGTTCAAGGCTGGGCTATTGT-3′ and 5′-TTCAGCCAC TTTGATGCAGCTA-3′; human *HMGCR*, 5′-GGGAACCT CGGCCTAATGAA-3′ and 5′-CACCACGCTCATGAGTTT CCA-3′; human *LDLR*, 5′-GTGCTCCTCGTCTTCCTTTG-3′ and 5′-GCAAATGTGGACCTCATCCT-3′; human *HMGCS1*, 5′-CTCCCTGACGTGGAATGTCT-3′ and 5′-GAACTGTCTGC CCAGGTGAT-3′; human *SQLE*, 5′-GCTGTGCTTTCCAGAG ATGG-3′ and 5′-GGCATCAAGACCTTCCACTG-3′; mouse *Hmgcr*, 5′-CTTGTGGAATGCCTTGTGATTG-3′ and 5′-AGC CGAAGCAGCACATGAT-3′; mouse *Ldlr*, 5′-AGGCTGTGGGC TCCATAGG-3′ and 5′-TGCGGTCCAGGGTCATCT-3′; mouse *Hmgcs*, 5′-GCCGTGAACTGGGTCGAA-3′ and 5′-GCATATAT AGCAATGTCTCCTGCAA-3′; mouse *Sqle*, 5′-AAATCAGAG CCGTGGGCTAC-3′ and 5′-GGAAGTGACACAGTTCTATG-3′. Ct values of target genes were normalized to the Ct values of a housekeekping control gene (18s, 5′-CGCGGTT CTATTTTGTTGGT-3′ and 5′-TCGTCTTCGAAACTCCGACT-3′) using the ΔΔCt method (Livak and Schmittgen, 2001) and expressed as relative mRNA expression levels compared to the control group which is arbitrarily set as 1.

### Protein Extraction, Immunoprecipitation, and Western Blotting

Whole cell lysates were obtained by re-suspending cell pellets in RIPA buffer (150 mM NaCl, 50 mM Tris pH 7.4, 0.1% SDS, and 1 mM EDTA) with freshly added protease inhibitor tablet (Roche, cat# 11697498001). Immunprecipitation was performed essentially as previously described (Li et al., 2018d). Briefly, anti-Brg1 (Santa Cruz, cat# sc-17796), anti- SREBP2 (Proteintech, cat# 28212-1), or pre-immune IgGs (Santa Cruz, cat# sc2027) were added to and incubated with ∼500 μg of cell lysates overnight before being absorbed by Protein A/G-plus Agarose beads (Santa Cruz, cat# sc-2003). Precipitated immune complex was released by boiling with 1X SDS electrophoresis sample buffer (0.05M Tris, 0.1M DTT, 2% SDS, 1.5 mM bromophenol blue, 1.1M glycerol). 50 μg of protein were loaded in each lane and separated by 8% PAGE-SDS gel with all-blue protein markers (Bio-Rad, cat# 1610373). For IP samples, 10% of the starting material was loaded as input. Proteins were transferred to nitrocellulose membranes (Bio-Rad, cat# 1620112) in a Mini-Trans-Blot Cell (Bio-Rad, cat# 1658004). The membranes were blocked with 5% fat-free milk powder at room temperature for half an hour and then incubated with the following primary antibodies at 4°C overnight. Western analyses were performed with anti-β-actin (Sigma, cat# A2228), anti-Brg1 (Santa Cruz, cat# sc-17796), anti-SREBP2 (Proteintech, cat# 28212-1), and anti-TBP (Proteintech, cat# 22006-1). Image J software was used for densitometrical quantification and densities of target proteins were normalized to those of β-actin or TBP. Data are expressed as relative protein levels compared to the control group which is arbitrarily set as 1.

### Chromatin Immunoprecipitation (ChIP)

ChIP assays were performed essentially as described before (Ernst et al., 2018; Kim et al., 2018; Ko et al., 2018; Nestal de Moraes et al., 2018; Pavlaki et al., 2018; Pellicelli et al., 2018; Rashid et al., 2018; Thiyagarajan et al., 2018; Wang et al., 2018; Zhao et al., 2018; Choi et al., 2019; Fan et al., 2019; Federico et al., 2019; Han et al., 2019; Holmes et al., 2019; Karanyi et al., 2019; Kong et al., 2019a,b; Li et al., 2019a,b,c,d,e; Liu et al., 2019b; Lu et al., 2019; Nishikawa et al., 2019; Paccez et al., 2019; Shao et al., 2019; Weng et al., 2019; Xu et al., 2019; Yang et al., 2019a) using an EZ-Magna ChIP kit (Millipore, cat# 17-10086). Briefly, cells (1 × 10^7^/∼10 reactions) were cross-linked with 1% freshly prepared formaldehyde at room temperature for 10 min. Cells were washed with PBS and re-suspended in cell lysis buffer and then nuclear lysis buffer to extract chromatin per vendor’s instruction. The resulting material was then sonicated to create appropriately sized (200–500 bp) chromatin fragments using a Bioruptor (Diagenode). For liver tissue ChIP, we used a Magna ChIP G Tissue kit (Millipore, cat# 17-20000). Briefly, chop tissue into small pieces (1–2 mm^2^) with a razor blade or scalpel. Transfer tissue into a tube with a screw cap lid and add formaldehyde to a final concentration of 1% and rotate tube at room temperature for 10 min. Chromatin was prepared by re-suspending fixed tissue pellet in tissue lysis buffer supplied by the vendor and sonicated to 200–500 bp. Aliquots of lysates containing 200 μg of protein were used for each immunoprecipitation reaction with the following antibodies: anti-Brg1 (Santa Cruz, cat# sc-17796), anti-acetyl histone H3 (Millipore, cat# 06-599), anti-acetyl histone H4 (Millipore, cat# 06-866), anti-trimethyl histone H3K4 (Millipore, cat# 07-473), anti-KDM3A (Proteintech, cat# 12835-1), and anti-SREBP2 (Abcam, cat# ab112046). For Re-ChIP, immune complexes were eluted with the elution buffer (1% SDS, 100 mM NaCO_3_), diluted with the re-ChIP buffer (1% Triton X-100, 2 mM EDTA, 150 mM NaCl, 20 mM Tris pH 8.1), and subjected to immunoprecipitation with a second antibody of interest. Precipitated DNA was amplified with the following primers: human *HMGCR* promoter, 5′-GACCAATAGGCAGGCCCTAGTGC-3′ and 5′-CTCTGCAG GGCCAAGAACAGG-3′; human *LDLR* promoter, 5′-TCCTC TTGCAGTGAGGTGAA-3′ and 5′-TTTCTAGCAGGGGGA GGAGT-3′; human *HMGCS1* promoter, 5′-TGGCCCGC ATCTCCTCTCAC-3′ and 5′-GCTAGGATTTTCCCTCGTG-3′; human *GAPDH* promoter, 5′-GGGTTCCTATAAATACGGA CTGC-3′ and 5′-CTGGCACTGCACAAGAAGA-3′; mouse *Hmgcr* promoter, 5′-TCGTGACGTAGGCCGTCAG-3′ and 5′-CCAATAAGGAAGGATCGTCCG-3′; mouse *Ldlr* promoter, 5′-AGCTTCAGGGGTTAAAAGAG-3′ and 5′-CGGTGCTCA TCCTTAGCTT-3′; mouse *Hmgcs* promoter, 5′-ATTGGTC GGAGAACCTCTC-3′ and 5′-AGGGGTGGGAACAAAGTCC-3′; mouse *Gpadh* promoter, 5′-ATCACTGCCACCCAGA AGACTGTGGA-3′ and 5′-CTCATACCAGGAAATGAGCTTGA CAAA-3′. 10% of the starting material was included as the input. Data are normalized to the input and expressed as % of recovery.

### Statistical Analysis

Data are presented as mean ± SD. For experiments concerning multiple groups, one-way ANOVA with *post hoc* Scheffe analyses were performed to evaluate the differences using an SPSS package (IBM analytics). The differences between two (control and experimental) groups were determined by two-sided, unpaired Student’s *t*-test.

## Results

### Down-Regulation of Cholesterogenic Gene Expression in Hepatocyte-Specific Brg1 Knockout Mice

**FIGURE 1:**
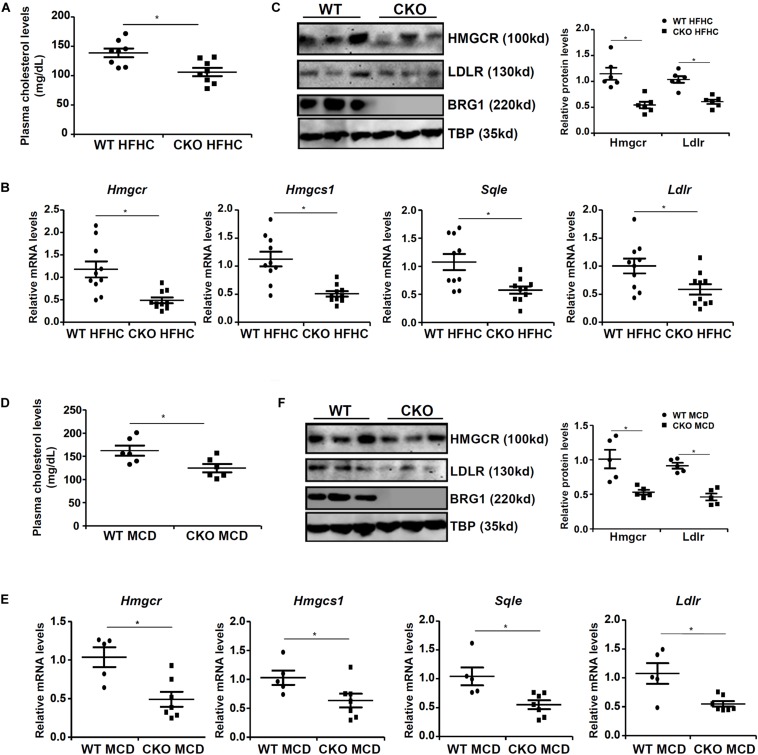
Down-regulation of cholesterogenic gene expression in hepatocyte-specific Brg1 deletion mice. **(A–C)** Wild type (WT) and hepatocyte-specific Brg1 knockout (CKO) mice were fed a high-fat high-carbohydrate diet (HFHC) for 16 weeks. **(A)** Hepatic total cholesterol levels. Expression of cholesterogenic gene expression was examined by qPCR **(B)** and Western **(C)**. Each lane represents a liver sample collected from a separate mouse. **(D–F)** Wild type (WT) and hepatocyte-specific Brg1 knockout (CKO) mice were fed a methionine-and-choline deficient diet (MCD) for 16 weeks. **(D)** Hepatic total cholesterol levels. Expression of cholesterogenic gene expression was examined by qPCR **(E)** and Western **(F)**. Each lane represents a liver sample collected from a separate mouse. Error bars represent SD. *p < 0.05 (one-way ANOVA with *post hoc* Scheffe test).

We first assessed the effect of BRG1 deficiency on cholesterol synthesis *in vivo* in two classical models of steatosis. BRG1 was specifically deleted from hepatocytes by Alb-Cre driven removal of the floxed *Smarca4* allele (Li et al., 2018a). In the first model, conditional BRG1 knockout (CKO) and wild type (WT) littermates were placed on a high-fat high-carbohydrate (HFHC) diet for 16 weeks. Compared to the WT mice, CKO mice exhibited significantly lower levels of cholesterol in the plasma (Figure 1A). In accordance, expression levels of several enzymes involved in the cholesterol biosynthesis pathway, including 3-hydroxy-3-methylglutaryl-CoA reductase (*Hmgcr*), low-density lipoprotein receptor (*ldlr*), 3-hydroxy-3-methylglutaryl-CoA synthase 1 (*Hmgcs1*), and squalene monooxygenase (*Sqle*), were all down-regulated in the livers of CKO mice than WT mice (Figures 1B,C). In the second model, the CKO mice and the WT mice were fed a methionine-and-choline-deficient (MCD) diet for 8 weeks to induce steatosis. BRG1 deficiency reduced plasma cholesterol levels and repressed expression of rate-limiting enzymes in the liver (Figures 1D–F).

**FIGURE 2:**
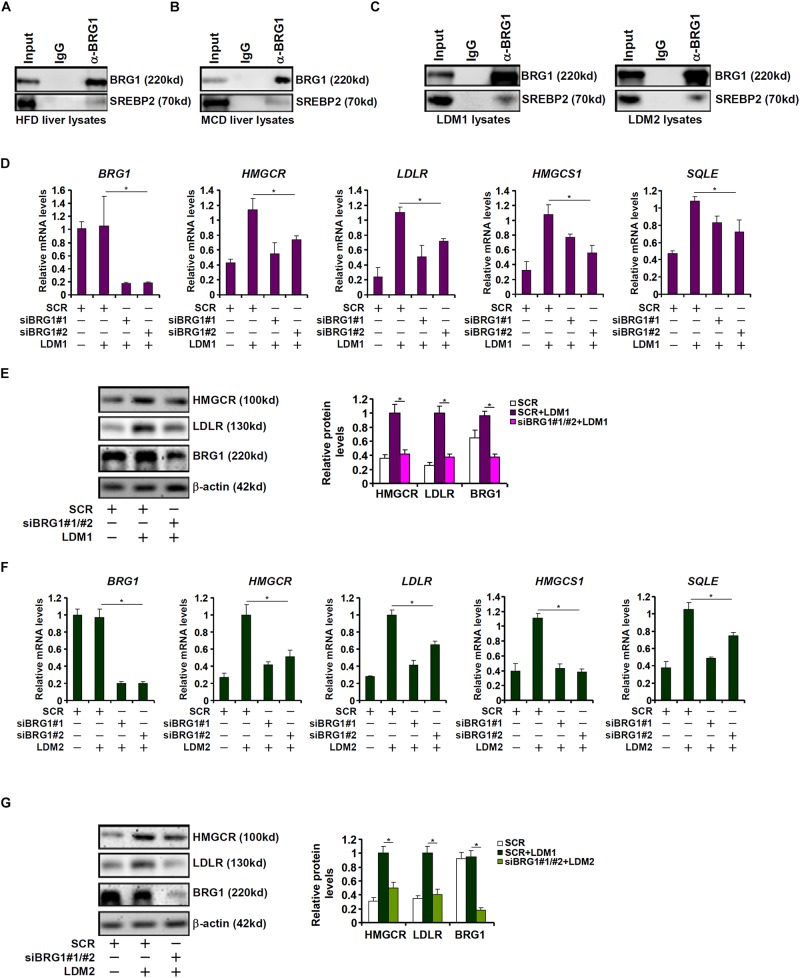
Down-regulation of cholesterogenic gene expression in Brg1-deficient hepatocyte. **(A)** C57/BL6 mice were fed an HFHC diet for 16 weeks. Nuclear lysates were extracted from the livers and co-immunoprecipitation was performed with indicated antibodies. **(B)** C57/BL6 mice were fed an MCD for 8 weeks. Nuclear lysates were extracted from the livers and co-immunoprecipitation was performed with indicated antibodies. **(C)** HepG2 cells were cultured in LDM1 or LDM2 for 24 h. Nuclear lysates were extracted and co-immunoprecipitation was performed with indicated antibodies. **(D,E)** HepG2 cells were transfected with small interfering RNA against BRG1 (siBRG1) or scrambled siRNA (SCR) and exposed to lipid-depletion media 1 (LDM1). Expression of cholesterogenic gene expression was examined by qPCR and Western. **(F,G)** HepG2 cells were transfected with siBRG1 or SCR and exposed to lipid-depletion media 2 (LDM2). Expression of cholesterogenic gene expression was examined by qPCR and Western. Error bars represent SD. *p < 0.05 (one-way ANOVA with *post hoc* Scheffe test).

Cholesterol synthesis at the transcriptional level is programmed by the transcriptional factor SREBP2 (Horton et al., 2002b). The observation that BRG1 deficiency in hepatocytes resulted in SREBP2-dependent cholesterogenic gene transcription prompted us to investigate the potential interplay between these two factors. Co-immunoprecipitation assays performed with liver nuclear lysates derived from either the high-fact diet (HFD) fed mice (Figure 2A) or the MCD fed mice (Figure 2B) showed that BRG1 formed a complex with SREBP2. Similar experiments performed with nuclear lysates extracted from LDM1/LDM2 treated hepatocytes confirmed that SREBP2 and BRG1 were in the same complex (Figure 2C).

SREBP2 activity can be modulated by cellular lipid levels. To this end, HepG2 cells were exposed to culture media containing lipid-depleted fetal bovine serum (LDM1). Exposure to LDM1 significantly up-regulated the transcription of cholesterogenic genes; BRG1 knockdown by two separate pairs of siRNAs attenuated the induction of cholesterogenic genes (Figures 2D,E). Alternatively, the cells were exposed to culture media containing a mixture of insulin-transferin-selenium (LDM2). LDM2-induced up-regulation of cholesterogenic genes was similarly weakened by BRG1 silencing (Figures 2F,G). Collectively, we conclude that BRG1 might contribute to hepatic cholesterol synthesis by acting as a regulator of pro-cholesterogenic transcription in hepatocytes.

### BRG1 Interacts With SREBP2

**FIGURE 3:**
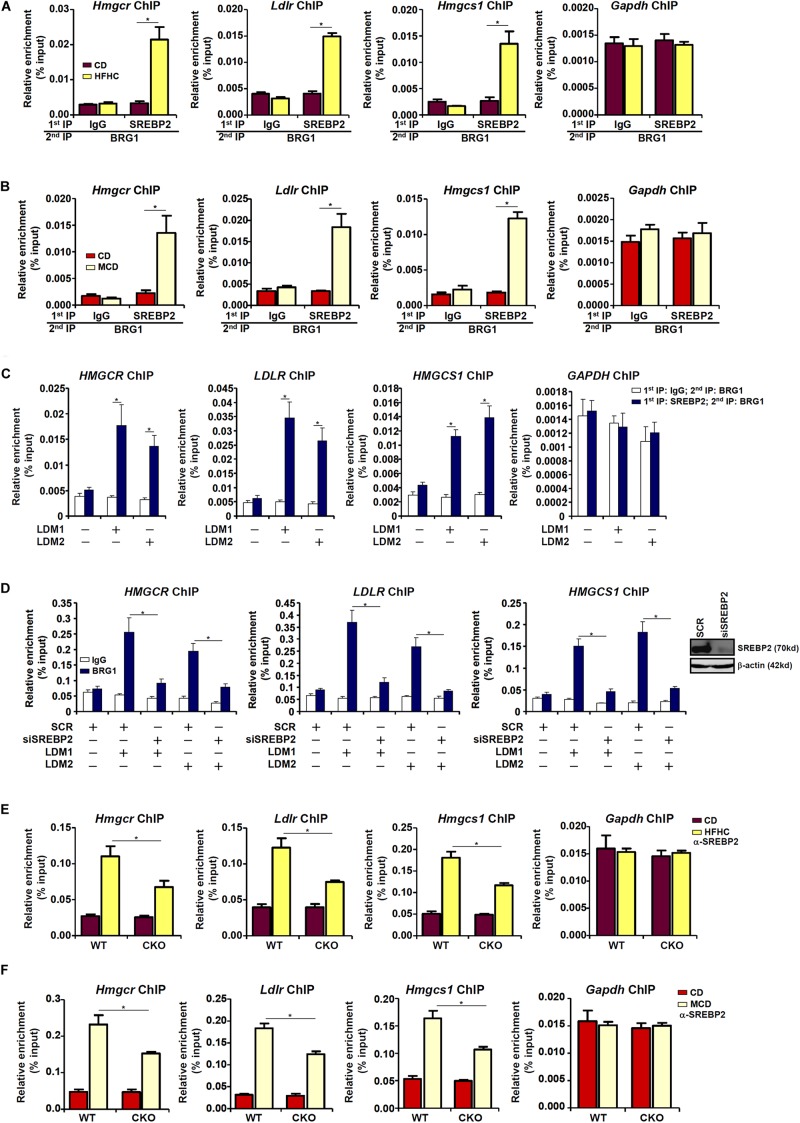
Dynamic interaction between Brg1 and SREBP2. **(A)** C57/BL6 mice were fed an HFHC diet for 16 weeks. Nuclear lysates were extracted from the livers and Re-ChIP was performed with indicated antibodies. **(B)** C57/BL6 mice were fed an MCD for 8 weeks. Nuclear lysates were extracted from the livers and Re-ChIP was performed with indicated antibodies. **(C)** HepG2 cells were cultured in LDM1 or LDM2 for 24 h. Nuclear lysates were extracted and Re-ChIP was performed with indicated antibodies. **(D)** HepG2 cells were transfected with siRNA targeting SREBP2 or SCR and exposed to LDM1 or LDM2. ChIP assays were performed with anti-BRG1. Inset, knockdown efficiency. **(E)** WT and CKO mice were fed an HFHC diet or 16 weeks. Nuclear lysates were extracted from the livers and Re-ChIP was performed with indicated antibodies. **(F)** WT and CKO mice were fed an MCD diet or 8 weeks. Nuclear lysates were extracted from the livers and Re-ChIP was performed with indicated antibodies. Error bars represent SD. *p < 0.05 (one-way ANOVA with *post hoc* Scheffe test).

Next, we performed Re-ChIP experiment to evaluate whether BRG1 interacted with SREBP2 on the cholesterogenic gene promoters. Using liver nuclear lysates from either the HFD fed mice (Figure 3A) or the MCD fed mice (Figure 3B), we found that there was a much stronger BRG1-SREBP2 interaction on the cholesterogenic gene promoters, but not the *Gapdh* promoter, in the steatotic livers than in the normal livers. Similarly, stronger BRG1-SREBP2 interaction was detected on the cholesterogenic gene promoters when HepG2 cells were stimulated with LDM1 or LDM2 (Figure 3C).

BRG1 occupancies on the cholesterogenic gene promoters were robustly augmented when the cells were exposed to LDM1/LDM2. The ability of BRG1 to occupy the cholesterogenic gene promoters was severely impaired when SREBP2 was depleted with siRNA in HepG2 cells (Figure 3D), confirming that BRG1 participates in regulating cholesterognic transcription by virtue of interacting with SREBP2. Reciprocally, the affinity of SREBP2 for its target promoters was decreased as a result of BRG1 deficiency in the liver (Figures 3E,F).

### Brg1 Regulates Dimethyl H3K9 Surrounding the Cholesterogenic Gene Promoters

**FIGURE 4:**
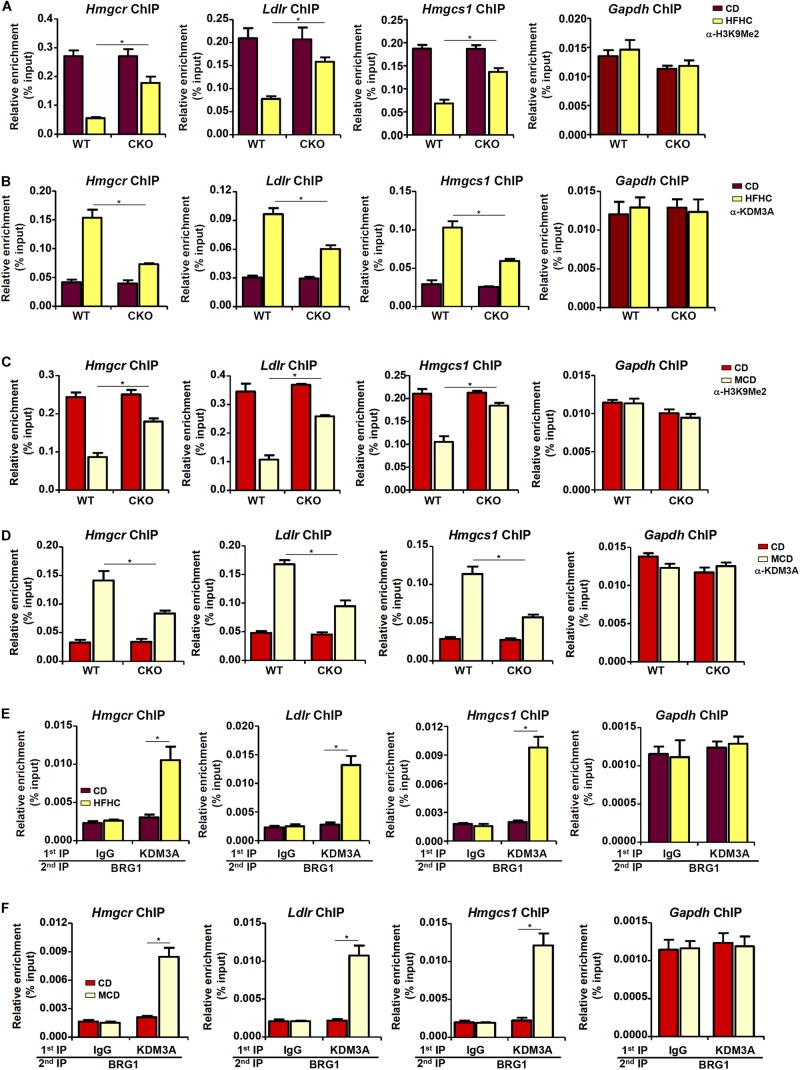
Brg1 regulates dimethyl H3K9 surrounding the cholesterogenic gene promoters *in vivo*. **(A,B)** WT and CKO mice were fed an HFHC diet or 16 weeks. Nuclear lysates were extracted from the livers and ChIP assays were performed with anti-dimethyl H3K9 and KDM3A. **(C,D)** WT and CKO mice were fed an MCD diet or 8 weeks. Nuclear lysates were extracted from the livers and ChIP assays were performed with anti-dimethyl H3K9 and KDM3A. **(E)** C57/BL6 mice were fed an HFHC diet for 16 weeks. Nuclear lysates were extracted from the livers and Re-ChIP was performed with indicated antibodies. **(F)** C57/BL6 mice were fed an MCD for 8 weeks. Nuclear lysates were extracted from the livers and Re-ChIP was performed with indicated antibodies. Error bars represent SD. *p < 0.05 (one-way ANOVA with *post hoc* Scheffe test).

The observation that BRG1 deficiency weakened the affinity of SREBP2 for its target promoters seems to suggest that BRG1 may contribute to the establishment of a more accessible chromatin structure for SREBP2 binding. We have previously shown that BRG1 can interact with the KDM3A, a histone H3K9Me2 demethylase, to erase the repressive histone marker dimethyl H3K9 from target promoters (Zhang et al., 2018; Shao et al., 2019). Therefore, we hypothesized that BRG1-meidated KDM3A recruitment to the cholesterogenic promoters may trigger the erasure of dimethyl H3K9 and facilitate SREBP2 binding. Consistent with this notion, ChIP assay showed that activation of cholesterogenic genes in the livers of HFHC-fed mice was mirrored by a concomitant loss of dimethyl H3K9, a repressive histone modification associated with closed (inaccessible) chromatin structure, surrounding the promoter regions (Figure 4A); BRG1 deficiency in hepatocytes, however, partially restored H3K9Me2 levels. Consistent with these data, there were increased occupancies of KDM3A to the cholesterogenic promoters *in vivo*; BRG1 deficiency significantly dampened KDM3A recruitment (Figure 4B). Similarly, accelerated cholesterol synthesis in the MCD-fed mice was accompanied by the removal of H3K9Me2 (Figure 4C) and the recruitment of KDM3A (Figure 4D), both of which relied on the presence of BRG1. More important, Re-ChIP assay demonstrated that the interaction between BRG1 and KDM3A on gene promoters was robustly stimulated by pro-cholesterogenic diets (Figures 4E,F).

**FIGURE 5:**
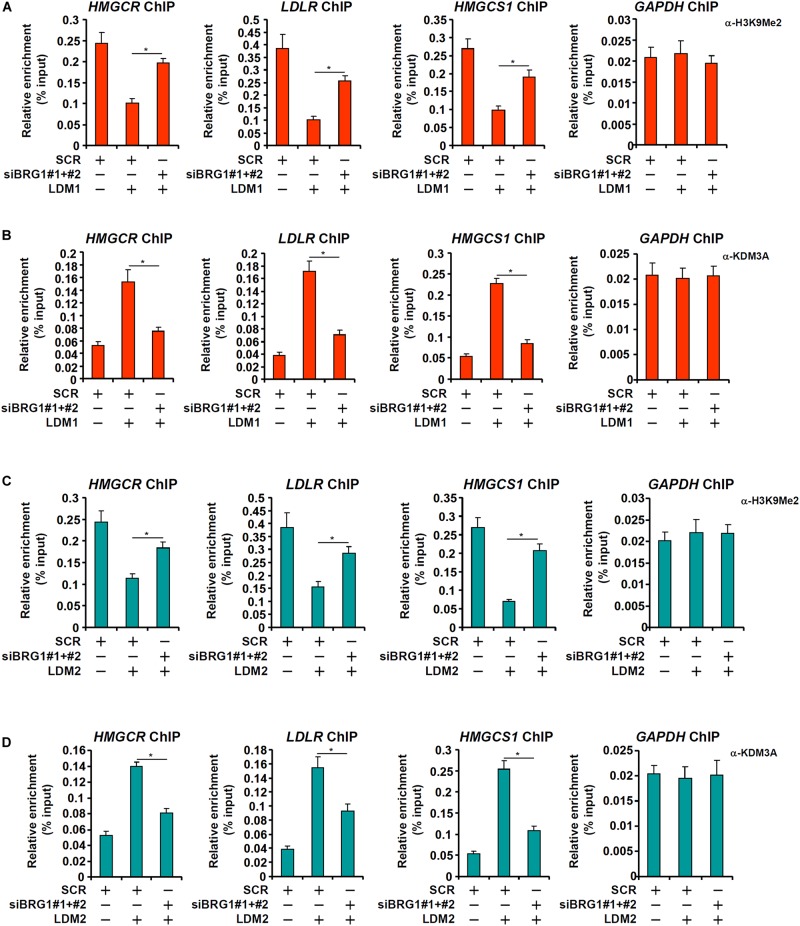
Brg1 regulates dimethyl H3K9 surrounding the cholesterogenic gene promoters *in vitro*. **(A,B)** HepG2 cells were transfected with small interfering RNA against BRG1 (siBRG1) or SCR and exposed to lipid-depletion media 1 (LDM1). ChIP assays were performed with anti-dimethyl H3K9 and KDM3A. **(C,D)** HepG2 cells were transfected with siBRG1 or SCR and exposed to lipid-depletion media 2 (LDM2). ChIP assays were performed with anti-dimethyl H3K9 and KDM3A. Error bars represent SD. *p < 0.05 (one-way ANOVA with *post hoc* Scheffe test).

In cultured hepatocytes, treatment with LDM1 (Figure 5A) or LDM2 (Figure 5C) led to the erasure of dimethyl H3K9 from the cholestrogenic promoters; BRG1 depletion, however, partially restored the levels of dimethyl H3K9. Consistent with these observations, LDM1 (Figure 5B) or LDM2 (Figure 5D) also stimulated the recruitment of KDM3A to the cholesterogenic promoters; BRG1 knockdown weakened the occupancies of KDM3A. Combined, these data suggest that an interaction between BRG1 and KDM3A might be responsible for epigenetic activation of cholesterogenic genes. Of note, an SREBP2-KDM3A complex was detected on the cholesterogenic promoters when the cells were exposed to LDM1 or LDM2 (Supplementary Figure S1). Similar to BRG1 depletion, depletion of SREBP2 also abrogated the recruitment of KDM3A and restored H3K9Me2 levels on the cholesterogenic promoters (Supplementary Figure S2).

### KDM3A Regulates the Transcription of Pro-cholesterogenic Genes in Hepatocytes

**FIGURE 6:**
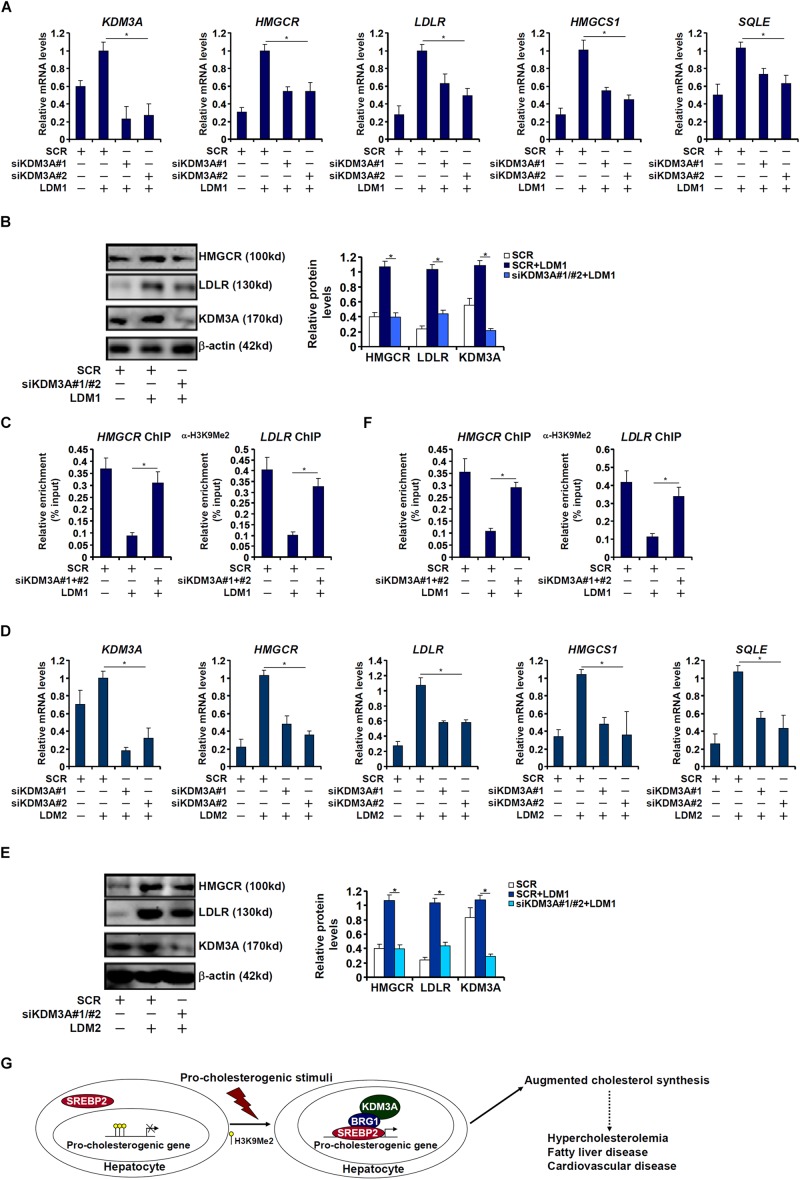
KDM3A regulates the transcription of pro-cholesterogenic genes in hepatocytes. **(A–C)** HepG2 cells were transfected with small interfering RNA against KDM3A (siKDM3A) or SCR and exposed to lipid-depletion media 1 (LDM1). Expression of cholesterogenic gene expression was examined by qPCR and Western. ChIP assay was performed with anti-dimethyl H3K9. **(D–F)** HepG2 cells were transfected with small interfering RNA against KDM3A (siKDM3A) or SCR and exposed to lipid-depletion media 2 (LDM2). Expression of cholesterogenic gene expression was examined by qPCR and Western. ChIP assay was performed with anti-dimethyl H3K9. Error bars represent SD. *p < 0.05 (one-way ANOVA with *post hoc* Scheffe test). **(G)** A schematic model. Under physiological conditions, high levels of dimethyl H3K9 on the cholesterogenic promoters keep the transcription of these genes at a relatively low level in hepatocytes. Upon exposure to a pathological stimulus, SREBP2 recruits BRG1 and KDM3A to remove the H3K9Me2 marker and augments the transcription rate of cholesterogenic genes. Increased cholesterol synthesis results in hypercholesterolemia and may contribute to the development of non-alcoholic fatty liver disease and cardiovascular diseases.

Finally, we asked whether KDM3A deficiency would influence the transcription of pro-cholesterogenic gene in hepatocytes. KDM3A silencing by siRNAs attenuated the activation of pro-cholesterogenic genes in hepatocytes exposed to LDM1 (Figures 6A,B) or LDM2 (Figure 6D,E). Consistent with its role as an H3K9 demethylase, KDM3A depletion restored the accumulation of dimethyl H3K9 on the pro-cholestrogenic promoters (Figures 6C,F). Therefore, we conclude that KDM3A may play an equally important role in determining the expression of pro-cholesterogenic genes in hepatocytes as BRG1.

## Discussion

Dys-regulation of cholesterol synthesis in hepatocytes is considered one of the key pathophysiological events in non-alcoholic fatty liver disease (Min et al., 2012). Augmentation of SREBP2 activity is the linchpin in cholesterol production (Shimano et al., 1997b). Previous studies have implicated BRG1, a chromatin remodeling protein, in the pathogenesis of non-alcoholic fatty liver disease (Li et al., 2018b; Fan et al., 2019; Liu et al., 2019a). Our new findings as summarized in this report demonstrate that BRG1 may contribute to cholesterol biosynthesis by functioning as a co-factor for SREBP2 to activate the transcription of cholesterogenic genes.

We have previously shown that BRG1 interacts with SREBP1c (Li et al., 2018b). Here we show that BRG1 can form a complex with SREBP2. Oliner et al. have demonstrated that SREBP1a and SREBP2 are similarly capable of interacting with co-factors (e.g., CBP) via the highly homologous N-terminal trans-activation domain of approximately 50 amino acids (Oliner et al., 1996). Structural comparisons of SREBP isoforms reveal that this trans-activation domain that both SREBP1a and SREBP2 possess is missing from SREBP1c due to alternative splicing (Horton et al., 2002b). It is generally agreed that SREBP factors, SREBP2 included, require co-regulators to occupy neighboring site(s) for optimal recognition and binding to the sterol response element. For instance, binding of Sp1 to a GC-rich region (known as repeat 3) downstream of the canonical SRE within the LDLR promoter is necessary for its trans-activation by SREBP2 (Sanchez et al., 1995). Similarly, CREB and NF-Y bind to the sequences flanking the sterol response element within the HMGCR promoter to facilitate SREBP2 recruitment and consequently trans-activation of the HMGCR promoter (Dooley et al., 1999). The genomewide co-localization of SREBP2 and other sequence-specific transcription factors on the chromatin has since been confirmed by ChIP-seq analysis (Seo et al., 2011). Because it has been previously shown that BRG1 can interact with Sp1 (Li et al., 2019d), CREB (Yang et al., 2006), and NF-Y (Oldfield et al., 2014), it remains to be determined whether BRG1 directly interacts with SREBP2 or an intermediate protein brokers the interaction.

One interesting finding is that BRG1 deficiency appears to dampen the affinity of SREBP2 for its target promoters. This observation is consistent with the notion that nascent chromatin structure is hostile to sequence-specific transcription factors and that stimuli-responsive chromatin remodeling is a prerequisite for target recognition and trans-activation. For instance, the class III deacetylase Sirt6 represses the transcription of cholesterogenic genes in hepatocytes by removing acetylation from H3K9 and H3K56 rendering an unfriendly chromatin micro-environment for SREBP2 to initiate transcription (Tao et al., 2013). Similarly, Kim et al. (2015) have demonstrated that SREBP2 preferably binds to the chromatin region with enriched trimethyl H3K4 and acetyl H3K9/K14 when activating target gene transcription. Although we focused on the levels of H3K9 methylation as a readout of chromatin status, it is noteworthy that BRG1 can potentially interact with and recruit multiple histone/DNA modifying enzymes including histone acetyltransferases (Yang et al., 2019b), histone methyltransferases (Weng et al., 2015), and cytosine dioxygenases (Li et al., 2019d). Therefore, the BRG1-dependent epigenetic landscape that contributes to SREBP2-mediated pro-cholesterogenic transcription may be more complicated than suggested by the current data and clearly warrants further investigation. Another caveat regarding the proposed model is that SREBP2, in addition to histones, may be directly subjected to modifications, which then fine-tune its activity. For instance, Ericsson and colleagues have argued that the histone acetyltransferase p300 can directly acetylate SREBP2 and that acetylation of SREBP2 regulates its stability (Giandomenico et al., 2003). Arito et al. (2008) have presented data to report that a SUMOylation and phosphorylation switch, in response to the growth factor IGF-1, of SREBP2 boosts its transcriptional activity and thus enhances cholesterol synthesis. Whether BRG1 is able to bridge post-translational modification of SREBP2 to regulate its activity is certainly a lingering issue awaiting clarification.

Our data suggest that BRG1 regulates transcription of cholesterogenic genes in part by recruiting the histone demethylase KDM3A. A natural question to ask is whether targeting KDM3A may be associated beneficial effects on metabolism. The Zhang laboratory has discovered that mice with systemic KDM3A deletion develop spontaneous obesity and hyperlipidemia largely due to a skewed metabolome in the adipose tissue (Tateishi et al., 2009), suggesting that targeting KDM3A globally to correct metabolic disorders may not be a desirable strategy. However, whether and, if so, how cholesterol synthesis in the liver might be impacted by KDM3A deficiency was not examined by the Zhang group and therefore remains unknown. At variance with the Tateishi paper (Tateishi et al., 2009) is another report by Nakatsuka et al. (2017) showing that KDM3A may promote hepatocellular carcinogenesis in mice and in humans. Since HCC is generally considered as the ultimate consequence of NASH without effective intervention, targeting KDM3A may be considered as a reasonable approach to treat NASH.

One of the major limitations regarding the present study is its focus on a single repressive histone modification (H3K9Me2). Previously Briggs and colleagues have reported that the lysine methyltransferase Set1, which is responsible for catalyzing the active trimethyl H3K4 marker, regulates the synthesis of ergosterol, the yeast equivalent of mammalian cholesterol (South et al., 2013). In addition, WDR5, a core component of the mammalian H3K4 methyltransferase complex, is detected on the HMGCR promoter and presumably activates HMGCR transcription (Vermeulen et al., 2007). We have previously that BRG1 is associated with an H3K4 methyltransferase activity (Weng et al., 2015) and can promote the deposition of trimethyl H3K4 on the target genes of SREBP1c (Li et al., 2018b). Whether a similar scenario applies to the current model as proposed (Figure 6G) awaits further investigation.

In summary, building on previous findings that BRG1 plays pivotal roles in regulating free fatty acid synthesis we present data to show that BRG1 functions as a co-factor for SREBP2 to regulate cellular cholesterol synthesis. Continuing this line of investigation with a focus on the molecular mechanism whereby BRG1 regulates SREBP2 activity will hopefully solidify the role of BRG1 leading to a rationalized decision to target BRG1 in the intervention of NASH.

## Data Availability Statement

The raw data supporting the conclusions of this article will be made available by the authors, without undue reservation, to any qualified researcher.

## Ethics Statement

The animal study was reviewed and approved by the Committee on Ethical Conduct of Animal Studies of Nanjing Medical University.

## Author Contributions

YX and XF conceived the project and supervised the project. ZF, MK, ML, and WH performed the experiments and collected the data, and analyzed the data. YX wrote the manuscript. ZF, XF, and YX secured the funding.

## Conflict of Interest

The authors declare that the research was conducted in the absence of any commercial or financial relationships that could be construed as a potential conflict of interest.
