# Supplementary material for: Brahma Related Gene 1 (Brg1) Regulates Cellular Cholesterol Synthesis by Acting as a Co-factor for SREBP2
Source: Front Cell Dev Biol. 2020 May 15;8:259. doi: 10.3389/fcell.2020.00259 (PMC7243037; doi:10.3389/fcell.2020.00259)
Supplement: Supplementary file 1 [file Data_Sheet_1.pdf]

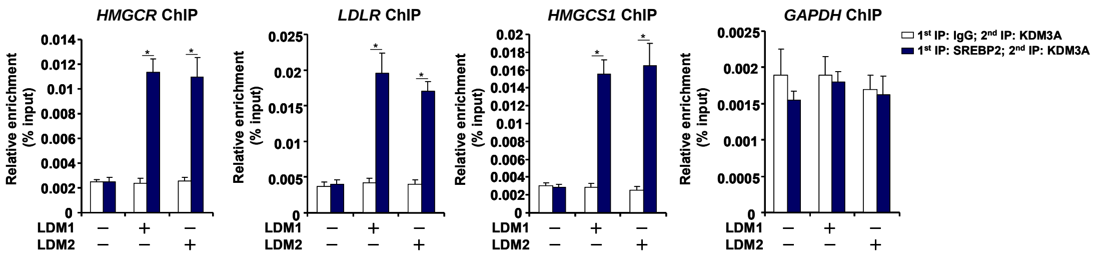

Figure S1: HepG2 cells were cultured in LDM1 or LDM2 for 24 hours. Nuclear lysates were extracted and Re-ChIP was performed with indicated antibodies.

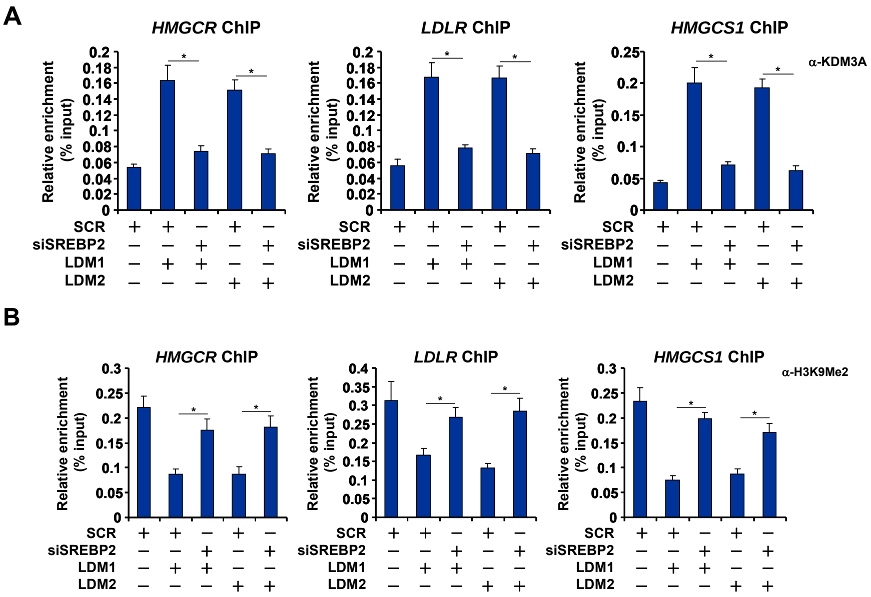

Figure S2: HepG2 cells were transfected with siRNA targeting SREBP2 or SCR and exposed to LDM1 or LDM2. ChIP assays were performed with anti-KDM3A (A) or anti-H3K9Me2 (B).
